# Hearing assessment in dental practitioners and other academic professionals from an urban setting

**DOI:** 10.1186/1746-160X-10-1

**Published:** 2014-01-18

**Authors:** Brita Willershausen, Angelika Callaway, Thomas G Wolf, Vicky Ehlers, Lukas Scholz, Dominik Wolf, Stephan Letzel

**Affiliations:** 1 Department of Operative Dentistry, University Medical Center of the Johannes Gutenberg University Mainz, Augustusplatz 2, 55131 Mainz, Germany; 2 Institute of Occupational, Social and Environmental Medicine, University Medical Center of the Johannes Gutenberg University Mainz, Kupferbergterrasse 17-19, 55116 Mainz, Germany

**Keywords:** Hearing assessment, Dental practitioners, Audiometric determination, Urban setting

## Abstract

**Introduction:**

Extended exposure to high-speed handpieces and other noise-intensive devices might put dentists at risk for possible hearing impairment. The aim of this study was to determine the hearing ability of dentists and other scientists for comparison.

**Methods:**

After approval by the ethics committee, 115 subjects (dentists and other academic professionals as controls) of both genders were enrolled in the study. Exclusion criteria were colds, ear-blockages or abnormal hearing-thresholds. An audiometric determination (Oscilla USB audiometer, AudioConsole 3, Inmedico A/S, Denmark) was performed in the frequency range of 125Hz to 8 kHz for both ears. Anamnestic data and number of years in the profession were assessed using a questionnaire. Differences between groups were analyzed with the Mann–Whitney-*U*-test.

**Results:**

Data from 53 dentists and 55 other academic professionals (69.4% male, 30.6% female) with a mean age of 51.7 ± 9.6 years and similar gender distributions in both groups were analyzed. The audiometric tests for the right and left air conduction showed that the hearing of dentists tended to be slightly more impaired than in the control subjects. For the frequencies 3 kHz and 4 kHz these differences were statistically significant for both ears. In contrast, no significant differences were found in this range for bone conduction.

**Conclusions:**

Hearing impairment in dentists was slightly higher than in controls. Although other factors like environmental noise exposure were comparable for both groups, occupational exposure to high-speed handpieces and other noisy devices can be an additional burden for the hearing.

## Introduction

All sounds, regarded as pleasant or unpleasant depending on the subjective experience, stimulate hearing in humans. The respective vibrational energy of the sound reflecting surfaces is captured as sound pressure through the ear canal at the ear drum. Noise is defined to consist mostly of undesirable tones or sounds, where these limits have to be regarded as very individual, because often certain frequencies are perceived subjectively by different individuals as sound or noise. Exposure to prolonged irregularly composed frequency ranges and raised noise levels are perceived as uncomfortable or even painful, and can cause numerous physical and psychological disorders or can even induce hearing loss [1-3]. Among the increased risks are for example those for cardiovascular diseases as well as for depression [4-6]. Due to environmental influences, street noise, train and air traffic as well as noise from industries or associated with various media and listening to music, noise pollution has become an everyday burden for all sections of the population [7]. In a recent representative survey from a European country with a high population density it was shown that noise was experienced as one of the most strongly perceived environmental disturbances. Traffic noise was rated as bothersome by 55% of the participants, 40% of the subjects listed noise from the neighbors as important causes for noise pollution, and a third of the population mentioned industry and trade as disturbing and irritating factors [8]. In the study by Lewis et al. [9] conducted in the same year, the risk for a permanent noise-induced hearing loss was estimated for a large urban U.S. setting. They found that in addition to noise pollution from occupational activities, exposure to noise from “non-occupational activities” (e.g. listening to MP3 players and stereos, mass transit use, attending concerts, use of lawn mowers etc.) added substantially to the risk for hearing impairment.

In general, acute damage to the hearing can be caused by constant exposure to sound pressure levels exceeding 85 dB, and by a sound pressure level exceeding 120 dB already after a few seconds. Also, extremely high sound pressure levels like from blasts or explosions in close proximity to the ear normally lead to permanent damage.

In their daily profession especially dentists and dental personnel are exposed to a noise level of different frequency ranges due to the use of high speed handpieces, various instruments and ultrasound devices [10-14]. The extent of possible noise levels was measured in 98 dental offices and nine dental laboratories in the city of Hamedan and maximum sound levels of 85.8 dB and 92.0 dB were found [10]. In an investigation with 32 dental students (mean age 26 years) in India it was even shown that the stay in dental clinics can lead to a small but consistent shift of hearing threshold [11]. In another study from Belgium 388 dentists from Flanders were questioned about potential occupational problems. Besides lower back pain (54%), vision problems (52.3%) and allergies (22.5%) also auditory disorders (19.6%) were mentioned. In addition, 13 dentists were observed over a period of 10 years, and especially for the left ear at 4 kHz a hearing reduction was found which, which could be indicative for a noise trauma [12].

The impairment by dental noises always depends on the frequency intensity, the daily intervals of the noise exposure, the daily treatment time and the years in the profession, the individual sensitivity and the distance from the respective instruments and devices. In numerous studies the impairment through noise was tested in dentists and the dental personnel, and possible consequences were assessed [15-18]. With the introduction of the high speed air turbine by the S.S. White Company in the year 1957, revolutions of up to 300,000 U/min were reached for the first time. This was quickly followed in 1959 by the first warnings about possible damages to the health, caused by the high frequency turbine noises and vibrations [19]. Already at that time, regular control assessments with audiograms were suggested for the dental team to prevent early damages from usage of the modern turbines and ultrasound devices. The new turbines lead to noise levels of above 84 dB, whereas the older belt-driven Doriot handpieces were with revolutions of 6,000 U/min considerably quieter. Since numerous studies [20-22] pointed towards a possible damage to the hearing of dentists, in 1974 the American Dental Association (ADA) acknowledged that the frequent usage of high frequency cutting instruments could lead to hearing impairments. In later studies the consequences of noise exposure in dentists were assessed for possible health risks. In others studies, however, a possible relation of the usage of high frequency turbines and damage to the dentists’ hearing was rather critically discussed and considered as an uncertain fact [14,23,24]. The aim of the present study was to assess the hearing abilities of dentists and other academic professionals to determine possibly significant differences in their hearing.

## Material and methods

A total of 115 subjects of both genders could be recruited for the present cross-sectional study to assess the hearing of dentists and other academic professionals. The subjects were alerted to participation in the free-of-charge study on hearing partly through flyers and partly by means of person-to-person communication. The project was introduced during the annual board meeting of the professional representation of the dentists from the state of Rhineland-Palatinate (Germany). All participants of the study lived and worked in the metropolitan Rhine-Main region, Germany, have worked in their profession for at least 10 years, and comprised an age range of 38 to 73 years. After the approval by the ethics commission of the professional representation of the dentists from the state of Rhineland-Palatinate (Nr. 837.439.11(7981)), a survey was conducted among dentists and other academic professionals (control subjects), comprising physicians, mathematicians, computer scientists, biologists and chemists, using a questionnaire, which provided, in addition to general anamnestic data, information about earlier or acute damage to the hearing or other ear disorders as well as about the years in the profession. Prior to an assessment of the hearing all subjects received detailed information about the audiometric tests, and only after having obtained a written consent from them, the subjects were enrolled in the study. All data and findings were evaluated anonymously.

By means of an extensive questionnaire, inclusion and exclusion criteria were determined for the participation in this hearing assessment. Among the inclusion criteria were for the dentists at least ten years working as dental practitioner; in addition, in all subjects no impaired hearing, no general disorders of the ear or the sound pathway as well as no hearing damages like tinnitus or acoustic hallucinations could be present and the subjects were to be free from common colds.

After filling in the questionnaires, all subjects participated in an audiometric test for both ears, using both air and bone conduction. The tests for the determination of the subjective hearing took place in shielded rooms. By means of the audiometer (Oscilla® USB audiometer, AudioConsole 3, Inmedico A/S, Denmark) of the Institute of Occupational, Social and Environmental Medicine of the Johannes Gutenberg University Mainz, the hearing for the frequency range of 125 Hz to 8 kHz was assessed in the dental practitioners and the non-dental control subjects.

### Statistical analysis

The statistical analyses of the data were performed using the program STATA/IC 12.1 (StataCorp LP, College Station, TX, USA) in cooperation with the Institute of Occupational, Social and Environmental Medicine. Absolute and relative frequencies were given for the categorical variable gender, and means and standard deviations for the continuous variables age and intensities in dB. Differences in the variables between the two groups were analyzed using the Chi square-test (for the categorical variable gender) and the Mann–Whitney *U*-Test (for the continuous variables age and intensities in dB). A significance level of p < 0.05 was chosen.

## Results

**Figure 1:**
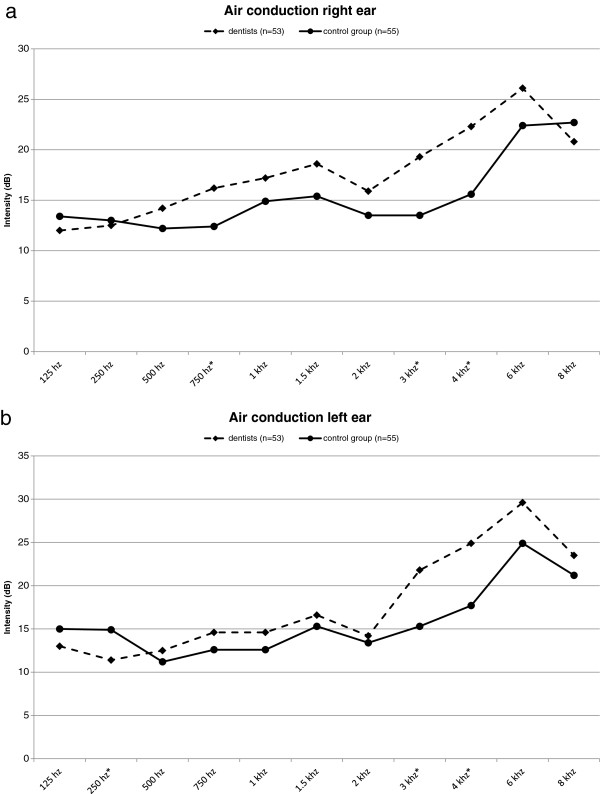
**Results of the audiometric tests for air conduction for the right and left ear.** The comparison of the results of the audiometric tests for air conduction for the dentists (n = 53) and the control group (n = 55) showed that the hearing impairment was only slightly more pronounced in the dentists than in the control subjects, at frequencies of 3 kHz and 4 kHz the differences were marginally statistically significant for the right **(a)** as well as the left ear **(b)**; asterisk: P < 0.05.

**Figure 2:**
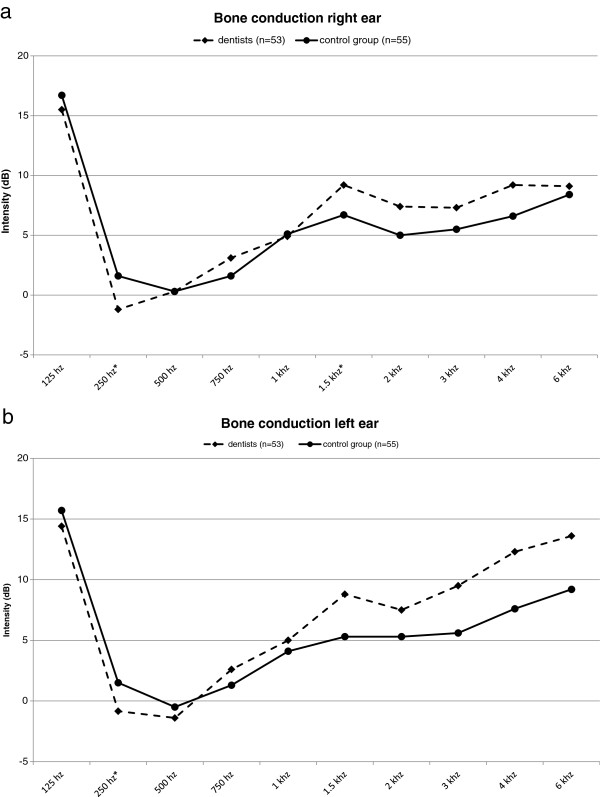
**Results of the audiometric tests for bone conduction for the right and left ear.** The comparison of the results of the audiometric tests for bone conduction of the dentists (n = 53) and the control group (n = 55) showed that nearly identical residual hearing was observed for the dentists and control subjects for the right ear **(a)** and the left ear **(b)**; asterisk: P < 0.05.

After the checking of all questionnaires and audiometric tests, the data from a total of 75 men and 33 women (53 dentists, 55 control subjects) could be evaluated. Subjects with prior ear disorders or presently suffering from hearing loss and those with incomplete or missing data were excluded from the study. In Table 1 the age and gender distributions are given for the entire study population and stratified according to study group. The mean age of the participants of the present study was 51.7 years (SD: 9.6 years, range: 34–74 years). The gender distribution in the two study groups was almost identical. The dentists were with a mean age of 53.5 years (SD: 9.4 years, range: 34–69 years) slightly older than the subjects in the control group, whose mean age was 50.0 years (SD: 9.6, range: 36–74 years). The results of the audiometric tests for the dentists and control subjects are depicted in Figures 1a, b for air conduction (left and right ear) and in Figures 2a, b for bone conduction (left and right ear).

**Table 1:** Gender and age distributions for the total study population (n = 108) and stratified for the study groups

	**Total**		**Dentists**		**Control group**	
	**n**	**%**	**n**	**%**	**n**	**%**
**Total**	108	100	53	100	55	100
**Gender**^**a**^						
Male	75	69.4	37	69.8	38	69.1
Female	33	30.6	16	30.2	17	30.9
**Age**^**b **^**(years old)**		51.7 ± 9.6^c^		53.5 ± 9.4^c^		50.0 ± 9.6^c^
≤39	12	11.1	5	9.4	7	12.7
40-44	16	14.8	4	7.6	12	21.8
45-49	18	16.7	9	17.0	9	16.4
50-54	20	18.5	9	17.0	11	20.0
55-59	19	17.6	11	20.8	8	14.6
60-64	13	12.0	9	17.0	4	7.3
≥65	10	9.3	6	11.3	4	7.3

The percentages are column percent.

^a^p =0.935 for Chi Square Test; ^b^p > 0.05 for *t*-Test.

^c^mean ± standard deviation.

When the results of the audiometric tests were analyzed using a direct comparison of the dental practitioners with the academics not from the dental profession, it became apparent that as a rule the hearing impairment was only slightly more pronounced in the dentists than in the control subjects. At frequencies of 3 kHz (left ear: p = 0.0442; right ear: p = 0.0207) and 4 kHz (left ear: p = 0.0442; right ear: p = 0.0496) these slight differences were marginally statistically significant for air conduction of the left as well as the right ear (Mann–Whitney-*U*-Test). However, nearly identical residual hearing was observed for dentists and control subjects for bone conduction of the left and right ear, representing the integrity of the inner ear, and the values were no longer statistically significant.

## Discussion

In addition to improvements in security standards governing the everyday professional life in most industrialized Western European countries, including Germany, together with a minimization of possible risks, nowadays there is a strong emphasis on the prevention of occupational diseases. Especially members of the dental profession are, due to inappropriate posture while working and neglecting to take rest breaks, frequently at risk to subsequent symptoms of musculoskeletal disorders [25,26]. Dentists and dental personnel complain in addition about an impairment of their hearing, and not infrequently do they fear possible hearing disturbances, which might be caused by the daily use of dental instruments like high-speed handpieces and sound-emitting instruments, lasting several hours over a period of many years [12]. The influence of a possible high environmental noise exposure on additional irritations to the ear has not been explicitly investigated in studies concerning the risk of hearing loss in dentists. In the present study all subjects came from an area with high noise pollution, and it was to be investigated if the environmental noise exposure might mask an occupational noise exposure to the hearing.

When the noise levels in dental teaching institutions were assessed by Kadanakuppe et al. [27] using precision meters, values of 64 to 97 dB were recorded. Due to the increased availability of many dental high frequency devices as well as their constant usage, noise levels can increase so that they can come close to the limits of the risk for hearing loss.

Setcos and Mahyuddin [4] also assessed noise levels in a dental laboratory and a dental clinic. By means of measurements at ear level and in two meters distance from the operator, they could show that all noise levels in the dental clinic lay below 85 dB. A daily noise exposure level limit of 85 dB is defined by the German Occupational Safety and Health Ordinance BK-Nr. 2301, regulating the protection of workers against the risk from exposure to noise and vibrations, a value, which if exceeded poses a risk for hearing damage. If this value is maintained at a constant level for an 8-hour work day, five days per week, with a weekly dose of a total of 40 hours at 85 dB, this way the maximal dose for a weekly exposure would be reached. In the dental practice, however, these limits are exceeded only for short periods of time, in contrast to individuals, who are constantly exposed to high values at their work place. For enrolment in the present study dentists with long years of experience in their profession were chosen to assess the influence of the noise exposure during daily routine treatments.

For a precise assessment of noise in a given situation, the noise exposure level is determined, which is standardized and documented according to DIN EN ISO 9612 (2009–09). Bali et al. [10] could show a hearing impairment in a total of 32 dentists aged 20 to 30 years, affecting the frequencies of 4 and 6 kHz for the left and 6 kHz for the right ear, which resembles the results from the present study with much older subjects (aged 34 to 68 years). Especially in older studies importance is given to a cause-effect-relationship of high-frequency handpieces to a decrease in hearing; this is surely due to the usage of the technical devices from that time and the generally lower environmental noise exposure. In the present study with subjects from an urban setting at 3 and 4 kHz a slightly increased hearing impairment could be shown in the dentists in comparison to the control group. When considering the technical progress in the development of high speed handpieces, it can be assumed that a steady reduction of noise emission has been achieved by the manufacturers, so that the comparability of recent studies with the results from earlier studies is limited [28]. Already in 1978, Forman-Franco et al. [29], who used an audiometric survey of 70 dentists, also failed to detect a decrease in the hearing thresholds of the dentists, neither in the speech nor in the high frequencies. In spite of the low hearing impairment found among the dentists, certain preventive measures should be taken, like e.g. keeping an appropriate distance from the patient, taking breaks from high noise exposure, regularly maintaining the dental equipment and possibly also using hearing protection devices [17,30]. Hearing deficits affecting the frequencies of 1, 2 and 4 kHz were found in the present study in dentists as well as in the academic control group. Such an age-induced hearing impairment, which has also been shown in epidemiological studies of occupational groups like military personnel and industrial workers after audiometric tests were performed, can therefore be confirmed [31].

In the present investigation a special emphasis was based on the fact that the study was conducted in a very densely populated area in Germany, which is located in close vicinity to one of the largest European airports as well as an extensive industrial area and road network.

Therefore, for the hearing assessment, it has to be considered in particular that environmental influences make up a significant part of the possible noise exposure, because the subjects of the study have been located in this area for at least 10 years. In addition, the dental treatment devices are subject to constant further developments, so that occupational damages to the hearing loss recede more and more into the background.

## Conclusions

It can be concluded from the results of this study that hearing impairment in dentists was slightly higher than in the control subjects, but besides occupational exposure to high-speed handpieces and other noisy devices the additional noise exposure in modern industrialized nations also has to be considered. Especially important in this context is environmental noise pollution in densely populated urban areas.

## Competing interests

The authors declare that they have no competing interests.

## Authors’ contributions

TGW, LS and DW carried out the study. AC and VE performed the statistical analysis. BW, TGW, VE, SL and AC conceived of the study, and participated in its design and coordination. All authors read and approved the final manuscript.
